# Bibliometric analysis of peer-reviewed literature in transgender health (1900 – 2017)

**DOI:** 10.1186/s12914-018-0155-5

**Published:** 2018-03-21

**Authors:** Waleed M. Sweileh

**Affiliations:** Department of Physiology, Pharmacology/Toxicology, Division of Biomedical Sciences, College of Medicine and Health Sciences, An-Najah National University, Nablus, Palestine

**Keywords:** Transgender, Health, Bibliometric analysis, Human rights

## Abstract

**Background:**

Transgender community is marginalized and under-researched. Analysis of peer-reviewed literature in transgender health is needed to better understand health needs and human rights of transgender people. Therefore, the aim of this study was to analyze global research activity in transgender health published in peer-reviewed journals.

**Methods:**

Peer-reviewed documents in transgender health were retrieved using Scopus database. VOSviewer was used to map frequently encountered author keywords while ArcGIS 10.1 was used to map the geographical distribution of the retrieved documents. Most active countries, institutions, and authors were presented. The study period was set from 1900 to 2017.

**Results:**

In total, 5772 peer-reviewed documents were obtained. English (5008; 86.8%) was the most frequently encountered language. A dramatic increase in the number of publications was seen in the last decade. The retrieved documents had an average of 12.1 citations per document and *h*-index of 92. Most frequently encountered author keywords were Human immunodeficiency virus infection and acquired immune deficiency syndrome (HIV/AIDS), mental health, and discrimination. Authors from 80 different countries contributed to publishing the retrieved documents. Publications originated mainly from Northern America, certain European countries, Australia, and Brazil. Professor Gooren, L.J.G. was the most active author in this field with 104 (1.88%) publications. Top active authors were in the fields of endocrinology, plastic surgery, psychiatry/psychology, public health, and sexology. Five of the top ten active authors were from the USA, three were from the Netherlands, and two were from Belgium. The most active institution was the *VU University Medical Center* (Netherlands) (184; 3.2%) followed by the *University of California, San Francisco* (USA) (157; 2.7%). The *International Journal of Transgenderism* was most active (284; 4.9%) in publishing articles in transgender health. However, documents published in the *American Journal of Public Health* had the highest impact with 53.5 citations per article.

**Conclusion:**

There was a noticeable growth of research in transgender health in the last decade. Researchers from different world regions need to get involved in health and human rights research of transgender community.

**Electronic supplementary material:**

The online version of this article (10.1186/s12914-018-0155-5) contains supplementary material, which is available to authorized users.

## Background

The term “transgender” describes a wide range of persons who are present across different cultures and countries [1]. The opposite term to transgender is “cisgender” which is a description for people whose gender identity matches the sex that they were assigned at birth [2]. Unfortunately, no global and updated data on the number of transgender persons are available, but there are approximately 1 million transgender adults living in the United States of America (USA) [3, 4] and approximately 9.0 million living in Asia and the Pacific regions [5, 6]. Estimates from different world regions indicate that the prevalence of transgender identity varies between 0.1% and 1.1% of reproductive age adults [7].

Transgender people face several social and health challenges such as violence, stigma, discrimination, social rejection, and inadequate specialized healthcare facilities [8, 9]. There are tremendous efforts in many developed countries to recognize the human rights of transgender persons [10–12]. Part of the action plan to endorse transgender community was to get researchers and academics engaged in research activity pertaining to health needs and challenges facing transgender persons. The scientific community can endorse the transgender community in several ways. For example, *The Lancet* had issued a series of comments and reports on transgender health that aimed to increase awareness and to promote better health services for the transgender community [8, 9, 13–18]. Several specific journals have been launched to endorse health and human rights of lesbian, gay, bisexual, and transgender people (LGBTQ). The letter “Q” in the LGBTQ refers to those who identify as queer or are questioning their sexual identity [19].

The Institute of Medicine considers transgender persons as neglected, marginalized, understudied, and in critical and urgent need of research regarding their health status and health needs [1, 8]. Transgender individuals are usually researched within LGBTQ community. However, transgender people are different from lesbians, gays, and bisexuals and might have different health and life experiences than gay and lesbians [20]. In light of the large numbers of transgender persons and the international efforts to endorse and strengthen human rights of neglected and marginalized groups, analysis of global research activity in transgender health becomes important [21–29]. Therefore, the aim of this study was to assess peer-reviewed literature in transgender health and present the results in bibliometric tables and maps. Research in transgender health is an essential component in recognition of this community and its health needs and health rights. The community of transgender individuals is seeking social support from all communities including those in academia and research scientist. Findings of the current bibliometric analysis could be used to advocate the human and health rights of transgender people particularly in countries with limited research activity in this field. Furthermore, bibliometric data obtained from the current study could be used by international health organizations and health activists to encourage politicians and health policymakers to adopt issues related to transgender health and include these issues in their political agendas and campaigns.

## Methods

### Database

In bibliometric studies, a large database is used to retrieve relevant documents for subsequent analysis. This study was conducted using SciVerse Scopus which has been used in several previously published bibliometric studies [30–37]. The choice of Scopus was based on the advantages it has over Web of Science and Pubmed [38]. The duration of this study was set from 1900 to 2017. Furthermore, no language restriction was made in retrieving the relevant documents. Articles indexed in Scopus must have an English abstract, and therefore, the relevancy of any retrieved document could be confirmed by reading the English abstract regardless of the original language of the document.

### Search strategy and inclusion criteria

Search strategy and keywords used were illustrated in **(**Additional file 1**)**. Keywords used were partially obtained from published systematic reviews in transgender health [9, 39, 40]. The search strategy was based on both title search and journal search. In the title search, all documents with the following keywords in the title were retrieved:

Title(enby or queer or transfeminine or genderqueer or bigender or pangender or genderfluid or agender or "transgender*" or "transsexual*" or "trans men" or "trans women" or "gender dysphoria" or "gender non-conform*" or "sex* reaassig*" or "gender reassign*" or transvest* or travesty or "koti" or "hijra" or "mahuvahine" or "mahu" or "waria" or katoey or "cross dresser" or "bantut" or "nadleehi" or "berdache" or "xanith" or "gender dysphori*" or "gender incongruen*" or "gender non-conform*" or "gender affirm* surg*" or "gender variant" "FTM individ*" or "MTF individ*" or transgender* or "trans men" or transmen or transman or "trans man" or"trans male" or transmale or "trans women" or transwomen or transwoman or "trans woman" or "trans female" or transfemale or "trans masculine" or transmasculine or transsex* or transvest* or "sex reassignment" or "gender reassignment" or "gender change" or "sex change")) or((Title("gender minorit*") and title-abstract-key(transgender* or transsex*))) or ((Title("female to male" OR "male to Female" or M2F or F2M) and title-abstract-key(transgender or transsex*))) or ((Title("two spirits" or "non binary" or cisgender) and title-abstract-key (transgender or transsexual*)))

In journal search, all documents published in journals having the keyword “transgender” were retrieved under the condition that the document has the term transgender or transsexual in the title and/or abstract. The search looked like this: (Journal name (transgender*) and title-abstract-key (transgender or transsex* OR M2F or F2M)).

The results obtained from the strategies stated above were combined.

### Exclusion criteria

1. Documents published in non-health related journals such as “English teaching” or “English lite*” or Estudios or literature or geography were excluded.
2. Documents within the subject areas of computer, mathematics, physics, chemistry engineering, material science, agriculture, and veterinary were also excluded.
3. Documents with keywords pertaining to plants or animals or birds such as plant or flower or animal or fly or birds or vet* or butterf* were also excluded.
4. Only documents published in peer-reviewed journals were retrieved and analyzed. Conference abstracts/papers published in peer-reviewed journals were included in the analysis while conference abstract not published in peer-reviewed journals were not included in the study. Books and book chapters were excluded.

The asterisk is used in the search strategy as a wildcard to retrieve any possible related keyword while the quotation marks were used to retrieve the exact phrase written within the quotation.

### The validity of search strategy

In this study, the validity of the search strategy was confirmed by two methods. In the first method, the author manually reviewed the top 200 cited articles to make sure that all these articles were relevant to the theme of the study. In the second method, the number of articles for each of the top ten active authors was retrieved manually and compared with the number obtained through the search strategy. The results of the comparison showed strong and significant correlation (*r* = 0.96; *p* < 0.01) indicative of the high validity of research method.

### Bibliometric indicators

Retrieved documents were analyzed and results were presented as top ten active countries, authors, institutions, and journals. Data pertaining to active countries, journals, authors, and institutions are exported from Scopus to Excel program for tabulation. It should be noted here that Scopus counts the number of publications for each country based on author affiliation regardless of the position of the author in the author list. Therefore, there is a potential overlap in the number of publications assigned for each country. For example, a document with two authors from two different countries is counted once for each country. Same applies to data pertaining to most active authors and institutions.

### Bibliometric mapping

Author keywords were visualized using VOSviewer program [41] which is available for free download [42] along with its technical manual [43]. In VOSviewer, author keywords are mapped based on a minimum number of occurrences. The size of the node for each keyword in the map represents its frequency of occurrence in the retrieved documents. Larger node size indicated a high frequency of occurrence. VOSviewer can also be used to do overlay visualization in which keywords with yellow color represent the most recently used author keywords. The geographical distribution of publications was mapped using ArcGIS 10.1 software [44]. This software is available at the Faculty of Engineering at An-Najah National University and available for staff use. In ArcGIS, different colors are given to various world regions. Map regions with white color represent countries with missing data or no research contribution to the field being investigated. In the current study, the impact of publications was measured using Hirsch – index (*h*-index) which depends on the number of citations of the publication with higher *h*-index indicating higher impact [45].

## Ethical consideration

No human subjects were involved in this study and, therefore, no approval from institutional review board was required.

## Results

### Types of documents, languages, and subject areas

In total, 5772 documents were retrieved. The majority (4462; 77.3%) of retrieved documents were research articles followed by review articles (635; 11%), letters (209; 3.6%), notes (138; 2.4%), editorials (114; 2%), conference papers (73; 1.3%), and short surveys (65, 1.1%). At the time of writing this manuscript, there were 76 (1.3%) documents in press and were of unknown types. English was the main language (5008; 86.8%) of the retrieved documents followed by German (239; 4.1%), French (183; 3.2%), Spanish (88; 1.5%), Portuguese (69; 1.2%), and Polish (41; 0.7%). The majority of retrieved documents were published in journals indexed under the subject area of medicine (3436; 59.5%), followed by those indexed in social sciences (1849; 32.0%), psychology (1213; 21.0%), and nursing (337; 5.8%) Due to indexing of some journals in more than one subject area, the total percentage of subject areas exceeded 100%.

### Growth of publications

**Fig. 1:**
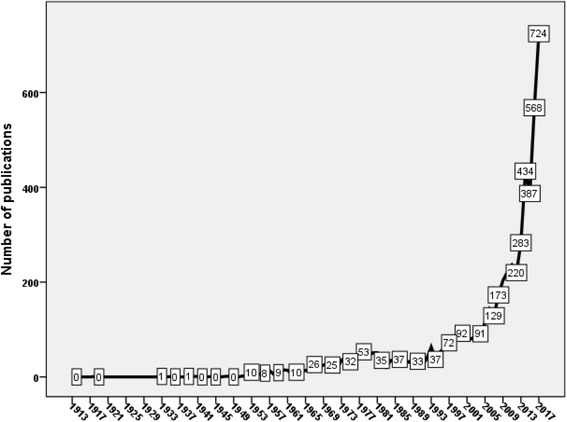
Annual growth of publications in transgender health (1900–2017)

The oldest published article in transgender health appeared in “*Zeitschrift für die gesamte Neurologie und Psychiatrie”* journal in 1913 [46]. This article discussed the neurological and psychiatric background of transvestism. The number of publications continued to grow slowly from 1913 to 2004 (Fig. 1). During this period the number of publications remained below 100 documents per year. A dramatic increase in the number of publications was observed after 2005. The maximum number of publications was recorded in 2017. The total number of documents published from 2005 to 2017was 3836 (58.5%). The growth rate of publications from 2015 to 2016 was 30.9% and that from 2016 to 2017 was 27.5%.

### Citation analysis

The retrieved documents received 69910 citations, an average of 12.1 citations per document. The *h*-index of the retrieved documents was 92. The range of citations obtained was from 0 to 524. The article that received the highest citations was published in *International Journal of Transgenderism* in 2012. This article provided clinical guidance for health professionals on how to assist transgender, transsexual, and gender non-conforming people in a safe and effective way [47]. Table 1 shows the list of highly cited articles. The list included seven research articles and three review articles. Three of the highly cited articles were published in *Journal of Homosexuality*. The list included articles that discussed aspects in the field of mental health, infectious diseases, endocrinology, neurology, and public health of the transgender community.

**Table 1:** Top 10 cited articles in transgender health

Title	Year	Source title	Cited by	Document Type
“Standards of Care for the Health of Transsexual, Transgender, and Gender-Nonconforming People, Version 7” [95]	2012	*International Journal of Transgenderism*	524	Article
“Endocrine treatment of transsexual persons: An endocrine society clinical practice guideline” [96]	2009	*Journal of Clinical Endocrinology and Metabolism*	458	Review
“A sex difference in the human brain and its relation to transsexuality” [97]	1995	*Nature*	430	Article
“HIV prevalence, risk behaviors, health care use, and mental health status of transgender persons: Implications for public health intervention” [98]	2001	*American Journal of Public Health*	412	Article
“Attempted suicide among transgender persons: The influence of gender-based discrimination and victimization” [99]	2006	*Journal of Homosexuality*	342	Article
“Estimating HIV prevalence and risk behaviors of transgender persons in the United States: A systematic review” [85]	2008	*AIDS and Behavior*	339	Review
“Gender violence: Transgender experiences with violence and discrimination” [100]	2001	*Journal of Homosexuality*	326	Article
“Suicide and suicide risk in lesbian, gay, bisexual, and transgender populations: Review and recommendations” [101]	2011	*Journal of Homosexuality*	304	Review
Worldwide burden of HIV in transgender women: A systematic review and meta-analysis [40]	2013	*The Lancet Infectious Diseases*	282	Article
Lesbian, gay, bisexual, and transgender-related content in undergraduate medical education [102]	2011	*Journal of the American Medical Association*	260	Article

### Mapping author keywords

**Fig. 2:**
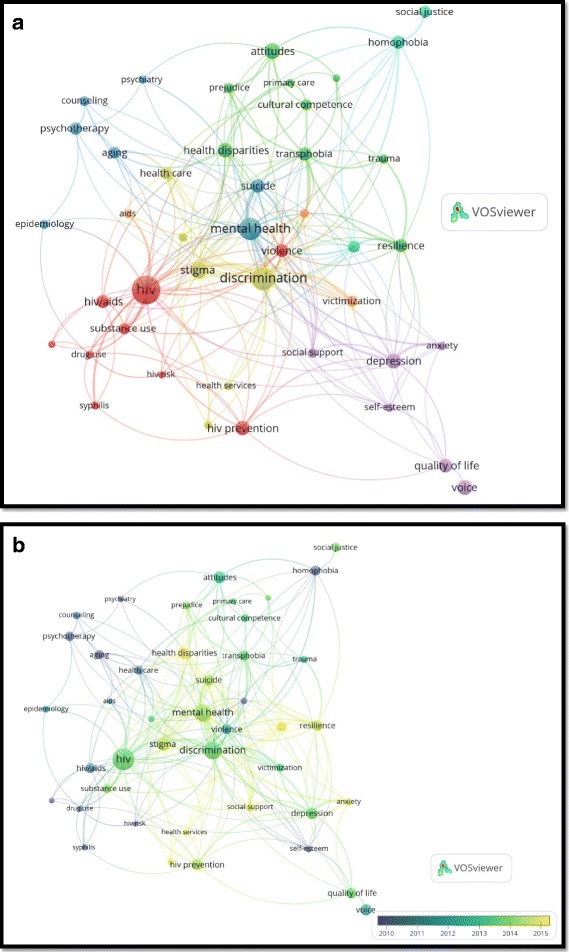
**a** and **b** Network visualization map and overlay visualization of author keywords in transgender health (1900–2017). These figures were created using VOSvViewer program which is available for free download from the Leiden University [42, 43]

Health-related author keywords with minimum occurrences of 10 were visualized and presented in network visualization map (Fig. 2 a). The most frequently encountered author keywords were grouped into seven different clusters shown in different colors. The most prominent clusters were those pertaining to HIV and other sexually transmitted diseases (STD), mental health, social acceptance and human rights, psychotherapy, and counseling. Overlay visualization, which shows the time of appearance of most frequently author keywords, indicated that keywords such as stigma, health disparities, health services, resilience, HIV prevention, and social support were the most recently used author keywords (Fig. 2 b).

### Geographical distribution of retrieved documents

**Fig. 3:**
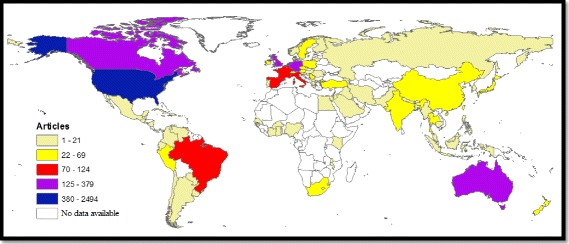
Geographical distribution of peer-reviewed documents in transgender health (1900–2017). This map was created using a copy of ArcGIS 10.1 program [44] available at the Faculty of Engineering at An-Najah National University. The author has full access to the Engineering Laboratories and to ArcGIS program

Authors from 80 different countries contributed to publishing the retrieved documents. Geographical distribution of the retrieved documents was based on the country affiliation of all authors participating in publishing the retrieved articles. Retrieved documents originated mainly from Northern America, certain European countries, Australia, and Brazil (Fig. 3). The African region, Middle East, and Eastern Europe had limited contribution to literature in transgender health.

### Highly active countries

The top ten active countries were shown in Table 2 along with the impact of their publications measured as the number of citations per article. Documents with authors from the Netherlands had the highest impact with an average number of citations of 35 per document followed by those from Belgium (17.1), and those from the USA (15.6). Authors in top ten active countries contributed to 4377 (75.1%) documents. Authors from the USA participated in publishing 2494 (43.2%) documents. The most active countries included one country from Latin America; Brazil (114; 2.0%). None of the countries in top ten list were from South – East Asia or Africa or the Middle East or Eastern Europe.

**Table 2:** Top ten active countries in transgender health research

Country	Frequency	% *N* = 5772	C	C/A
United States	2494	43.2	38982	15.6
United Kingdom	379	6.6	4231	11.2
Canada	321	5.6	4017	12.5
Germany	252	4.4	2431	9.6
Netherlands	247	4.3	8585	34.8
Australia	211	3.7	1968	9.3
France	124	2.1	551	4.4
Belgium	121	2.1	2069	17.1
Brazil	114	2.0	689	6.0
Spain	114	2.0	990	8.7

C: number of citations

C/A: number of citations per article

### Analysis of publication from the US

Further analysis of publications by US researchers indicated that authors from the East coast of the USA (Maine, New Hampshire, Massachusetts, Rhode Island, Connecticut, New York, New Jersey, Delaware, Maryland, Virginia, North Carolina, South Carolina, Georgia, and Florida) participated in publishing 810 (32.5%) documents while those from the West coast (California, Oregon, and Washington) participated in publishing 760 (30.5%) documents. The contribution of authors from the Midwest region (Illinois, Indiana, Iowa, Kansas, Michigan, Minnesota, Missouri, Ohio, Nebraska, North Dakota, South Dakota, and Wisconsin) was 650 (26.1%) documents while those by authors from the Southwest region of the USA (Nevada, New Mexico, Utah, Arizona, Texas, and Colorado) was the least (230; 9.2%). It should be noted that there is a certain degree of overlap in the number of publications due to research collaboration.

### Authorship analysis and active institutions/organizations

Approximately 10,000 authors participated in publishing the retrieved documents, an average of 2.4 authors per document. Table 3 shows top ten active authors Professor Gooren, L.J.G. was the most active with 104 (2.4%) publications. The most active authors were in the fields of endocrinology, plastic surgery, psychiatry/psychology, sexology, and public health. Five of the top ten active authors were from the USA, three were from the Netherlands, and two were from Belgium. When retrieved data were analyzed for most active institutions/organizations, the *VU University Medical Center* in the Netherlands ranked first with 184 (3.2%) publications followed by the *University of California, San Francisco* (USA) with 157 (2.7%) publications. Seven institutions of the top ten list were in the USA, one was in Canada, one was in Belgium, and one was in the Netherlands (Table 4).

**Table 3:** Top ten active authors in the field of transgender health

Rank	Name	Frequency	% N = 5772	Affiliation
1st	Gooren, L.J.G.	104	1.8	*VU University Medical Center, Department of Endocrinology, Amsterdam, Netherlands*
2nd	Reisner, S.L.	62	1.1	*Fenway Institute, Boston, United States*
3rd	Bockting, W.	56	1.0	*New York State Psychiatric Institute, Division of Gender, New York, United States*
4th	Hage, J.J.	46	0.8	*Antoni van Leeuwenhoek Ziekenhuis, Department of Plastic and Reconstructive Surgery, Amsterdam, Netherlands*
5th	T’Sjoen, G.	43	0.7	*University Hospital of Ghent, Department of Endocrinology, Ghent, Belgium*
6th	Cohen-Kettenis, P.T.	35	0.6	*VU University Medical Center, Department of Medical Psychology and Medical Social Work, Amsterdam, Netherlands*
7th	De Cuypere, G.	30	0.5	*University Hospital of Ghent, Department of Sexology and Gender Problems, Ghent, Belgium*
8th	Garofalo, R.	26	0.5	*Children’s Memorial Hospital, Division of Adolescent Medicine, Chicago, United States*
9th	Operario, D.	25	0.4	*Department of Behavior and Social Sciences, Brown University, Providence, United States*
10th	Nemoto, T.	24	0.4	*Public Health Institute Oakland, Oakland, United States*

**Table 4:** Top ten active institutions/organizations in transgender health

Institution/organization	Frequency	% (N = 5772)	Country
VU University Medical Center	184	3.2	Netherlands
University of California, San Francisco	157	2.7	USA
Harvard Medical University	139	2.4	USA
University of California, Los Angeles	105	1.8	USA
University Hospital of Ghent	88	1.5	Belgium
University of Toronto	79	1.4	Canada
Johns Hopkins University	76	1.3	USA
Columbia University in the City of New York	68	1.2	USA
Children’s Hospital Boston	59	1.0	USA
University of Washington, Seattle	58	1.0	USA

### Most active journals

Retrieved documents were published in journals with different scopes. The top ten list included journals in the field of transgender /sexual medicine, public health, endocrinology, plastic surgery, and psychiatry (Table 5). The *International Journal of Transgenderism* ranked first with 280 (4.9%) documents. However, documents in transgender health published in the *American Journal of Public Health* received the highest number of citations per article (53) followed by those published in *Journal of Homosexuality* (27). The majority of top 10 active journals were in the field of sexual medicine. One journal was in the field of public health, one was in plastic surgery, and one in the field of AIDS. Eight of most active journals were based in the USA while two were based in Europe.

**Table 5:** Top ten active journals in publishing in documents in transgender health research

Name of the Journal	Frequency	% (*N* = 4417)	C	C/A	Country
International Journal of Transgenderism	280	4.9	3186	11.4	USA
Archives of Sexual Behavior	216	3.7	4994	23.1	USA
Journal of Sexual Medicine	86	1.5	1512	17.6	USA
Plastic and Reconstructive Surgery	79	1.4	1062	13.4	USA
Journal of Homosexuality	75	1.3	2048	27.3	USA
American Journal of Public Health	65	1.1	3475	53.5	USA
Journal of Gay and Lesbian Social Services	53	0.9	641	12.1	USA
AIDS And Behavior	50	0.9	929	18.6	Netherlands
LGBT Health	58	1.0	250	4.3	USA
Journal Of Lgbt Issues In Counseling	41	0.7	382	9.3	USA
Journal Of Sex Research	41	0.7	570	13.9	UK

C: number of citations

C/A: number of citations per article

## Discussion

In this study, we aimed to assess and analyze growth and research trends of peer-reviewed publications in transgender health. Publications in transgender health showed a sharp rise after 2005. Several factors could be cited to explain the sharp rise in the number of publications. Of particular importance is the appearance of publications in transgender health in highly prestigious journals such as *The Lancet* and the *American Journal of Public Health* which encouraged researchers in various disciplines to get involved in this research topic. A potentially second reason is the findings that the number of transgender people is larger than expected even in epidemiological studies and surveys in non-western countries [48, 49]. A third possible reason is the active role of human rights and transgender groups which argued against stigma and discrimination faced by the transgender community [10, 50–54]. Furthermore, transphobia led many researchers and health organizations to address this issue in international forums in order to support the health and human rights of the transgender community [55–59].

The current study showed that mental health research dominated the field of transgender health. Most mental health issues faced by transgender people are due to cultural intolerance, social stigma, discrimination, violence, and victimization [60–64]. The social stress and stigma created higher prevalence of depression and suicidal ideation in transgender individuals compared with cisgender people [65, 66]. Transgender individuals are subject to psychological stress [67] due to discrimination and social rejection. It should be emphasized that mental health issues in transgender persons are not due to transgender identity [9]. Studies indicated that a major cause of depression and suicidal ideation among transgender individuals is the stigma, trans-phobia, sexual assault, violence, and social de-evaluation [68, 69]. The World Health Organization (WHO) is considering declassification of transgender and sexual orientation from mental health disease classification [70]. Such move by WHO was supported by the scientific community [71, 72]. A key element in overcoming mental problems such as depression, anxiety, and stress in transgender persons is social support and acceptance [73, 74]. Despite discrimination, stigma, and victimization, the transgender community had shown remarkable extent of resilience and survival [66].

The current study showed that global contribution to transgender health research was limited to the regions of Americas, Europe, South-East Asia, and Western Pacific. It was not surprising that countries such as USA, Brazil, Philippines, and Thailand showed relatively high research activity. Unofficial reports indicated large numbers of transgender people in these countries. The number of publications from African and Mediterranean regions was limited. This could be attributed to the limited number of transgender people in both regions or due to limited research capacity in these regions. African region is considered the highest in terms of numbers of people with AIDS [75, 76] which could be due to the presence of infected female sex workers, lack of healthcare, and preventive measures but not due to transgender identity [77–79]. For the Mediterranean region, the cultural and religious background does not allow for sexual freedom and, therefore, data regarding transgender people, HIV, gay, lesbians, and bisexuals are relatively limited and might be inaccurate in that region [80–84].

The current study indicated that literature in transgender health discussed several aspects of STD including HIV/AIDS. A study carried out in the USA found that 28% of transgender individuals had HIV and 21% had other sexually transmitted diseases [40, 85]. Lack of preventive health practices and lack of screening are potential causes for the high prevalence of STD among transgender individuals [86–89].

Transgender health is a global issue and publications in this field is growing as a separate field or as part of research in LGBTQ community as an overall. The author did his best to collect all peer-reviewed documents in transgender health to endorse research and encourage other researchers and clinicians to get engaged in this field. However, the author acknowledges the fact that not all publications and health journals are indexed in Scopus and therefore, some publications in this field might be missed. This is a common limitation in bibliometric studies [31, 36, 90–93].

## Conclusion

The results of the current study showed (1) dramatic increase in the number of publications in transgender health in the last decade; (2) the bulk of literature in transgender health focused on HIV/AIDS, mental health, and discrimination; (3) regions of Africa, Middle East, and East Europe had the least research contribution to transgender health; (4) most active and leading authors and institutions in transgender health were restricted to northern America and Europe; and (5) *h*-index of peer reviewed documents in transgender health was less than 100 but relatively higher than that recorded for other subjects [34, 94].

## Additional file

Additional file 1: Research strategy in transgender health (1900–2017). (DOCX 13 kb)

## Abbreviations

HIV/AIDS: Human Immunodeficiency Virus / Acquired Immunodeficiency Syndrome
LGBTQ: Lesbian, gay, bisexual, transgender, and queer
WHO: World Health Organization
