# Supplementary material for: Bibliometric analysis of peer-reviewed literature in transgender health (1900 – 2017)
Source: BMC Int Health Hum Rights. 2018 Mar 21;18:16. doi: 10.1186/s12914-018-0155-5 (PMC5863490; doi:10.1186/s12914-018-0155-5)
Supplement: Supplementary file 1 — Research strategy in transgender health (1900–2017). (DOCX 13 kb) [file 12914_2018_155_MOESM1_ESM.docx]

**Bibliometric analysis of peer-reviewed literature in transgender health (1900 – 2017)**

**Waleed M. Sweileh**

**Corresponding author**

Professor Waleed M. Sweileh

E-mail: [waleedsweileh@yahoo.com](mailto:waleedsweileh@yahoo.com)

Tel: + 970-599-225906

Department of Physiology, Pharmacology/Toxicology,

Division of Biomedical Sciences,

College of Medicine and Health Sciences,

An-Najah National University,

Nablus,

Palestine

**Additional file 1**

**Research strategy and keywords**

| **Step** | **Search query with keywords** |
| --- | --- |
| **1** | Title(enby or queer or transfeminine or genderqueer OR bigender OR pangender OR genderfluid OR agender OR "transgender*" OR "transsexual*" OR "trans men" OR "trans women" OR "gender dysphoria" OR "gender non-conform*" OR "sex* reaassig*" OR "gender reassign*" OR transvest* OR travesty OR "koti" OR "hijra" OR "mahuvahine" OR "mahu" OR "waria" OR katoey OR "cross dresser" OR "bantut" OR "nadleehi" OR "berdache" OR "xanith" OR "gender dysphori*" OR "gender incongruen*" OR "gender non-conform*" OR "gender affirm* surg*" OR "gender variant" "FTM individ*" OR "MTF individ*" OR transgender* OR "trans men" OR transmen OR transman OR "trans man" OR "trans male" OR transmale OR "trans women" OR transwomen OR transwoman OR "trans woman" OR "trans female" OR transfemale OR "trans masculine" OR transmasculine OR transsex* OR transvest* OR "sex reassignment" OR "gender reassignment" OR "gender change" OR "sex change")) OR ((TITLE("gender minorit*") AND TITLE-ABS-KEY(transgender* OR transsex*))) OR ((TITLE("female to male" OR "male to Female" or M2F or F2M) AND TITLE-ABS-KEY(transgender OR transsex*))) OR ((TITLE("two spirits" or "non binary" or cisgender) AND TITLE-ABS(transgender))) |
| **2** | SRCTITLE(transgender*) AND TITLE-ABS-KEY(transgender OR transsex* OR M2F or F2M) |
| **3** | **Combine results of 1 and 2** |
| **4 (exclusion)** | AND NOT TITLE-ABS-KEY(plant OR flower OR animal OR fly OR birds OR vet* OR butterf*) AND NOT SRCTITLE( "Higher Education") )) AND NOT SRCTITLE(geograph*) AND NOT SRCTITLE("english teaching" or English lite*" or Estudios or literature") AND ( EXCLUDE ( SUBJAREA,"AGRI " ) OR EXCLUDE ( SUBJAREA,"COMP " ) OR EXCLUDE ( SUBJAREA,"ENGI " ) OR EXCLUDE ( SUBJAREA,"EART " ) OR EXCLUDE ( SUBJAREA,"MATH " ) OR EXCLUDE ( SUBJAREA,"MATE " ) OR EXCLUDE ( SUBJAREA,"CENG " ) OR EXCLUDE ( SUBJAREA,"CHEM " ) OR EXCLUDE ( SUBJAREA,"ENER " ) OR EXCLUDE ( SUBJAREA,"PHYS " ) OR EXCLUDE ( SUBJAREA,"VETE " ) ) |
| **5** | **Limit**: LIMIT-TO ( SRCTYPE,"j " ) AND LIMIT-TO(1900 – 2017) |
